# Recycling and Utilization of Polymers for Road Construction Projects: An Application of the Circular Economy Concept

**DOI:** 10.3390/polym13081330

**Published:** 2021-04-19

**Authors:** Muhammad Kashif Anwar, Syyed Adnan Raheel Shah, Hatem Alhazmi

**Affiliations:** 1 Department of Civil Engineering, Pakistan Institute of Engineering and Technology, Multan 66000, Pakistan; kashifanwar723@gmail.com; 2 National Center for Environmental Technology (NCET), Life Science and Environment Research Institute (LSERI), King Abdulaziz City for Science and Technology (KACST), Riyadh 11442, Saudi Arabia; halhazmi@kacst.edu.sa

**Keywords:** circular economy, recycling, waste plastics, polymers, performance, sustainability

## Abstract

Numerous environmental issues arise as a result of a linear economy strategy: reserves become scarce and end up in landfills and as greenhouse gases. Utilizing waste as a resource or shifting towards a circular economy are among the effective strategies for addressing these issues. To track this shift, appropriate measures that concentrate on sustainable development while taking practical contexts into consideration are required. In this paper, we utilize plastic wastes as a replacement for bitumen for reuse aiming at a circular economy. The use of plastic waste materials, i.e., plastic bottles (PET) and gas pipes (PE) in asphalt materials as a bitumen modifier was studied through series of experimental lab test methods. Marshall samples were prepared using a conventional Marshall method containing five different percentages (0%, 5%, 10%, 15%, and 20%) of plastic content by total weight of bitumen. Samples were tested after 1 and 30 days and the result shows that the stability of plastic-modified asphalt concrete was increased after 30 days, while still meeting standard criteria with plastic contents up to 20%. Moreover, the addition of waste plastic in road construction is a very effective strategy for reusing plastic waste, which also provides economic and social benefits for a sustainable approach to road pavements.

## 1. Introduction

**Figure 1:**
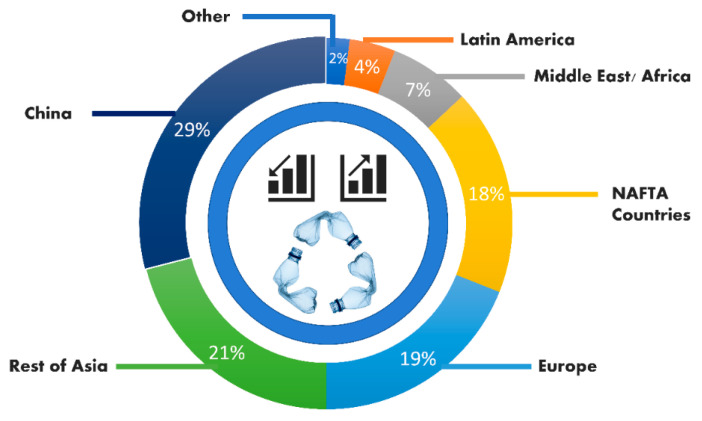
Plastics Statistics in The Oceans (2020).

Plastics are one of the present world’s largest innovations and widely used materials worldwide. Plastic demand has grown dramatically in the last 50 years, more than 322 Metric tons in 2015, with a projected doubling by 2035 [1,2,3]. According to an Australian plastics recycling study, overall use of plastics was 3.513 Million tons in 2016–2017, whereas only 0.42 Million tons were reused [4]. More than 30 million tons of plastic waste is generated in China annually [5]. In 2010, approximately 4.8 to 12.7 million metric tons of plastic were found in oceans [6]. The latest statistics of 2020 are presented in Figure 1. Asphalt mixtures have increasingly become an important material for roadway and airport pavement structures [7,8,9]. A kind of environmentally sensitive construction is flexible pavements. Pavement degradation can occur more quickly when environmental factors change adversely. Road authorities may incur extra costs to minimize the adverse changes in places where they will exist. If nothing is done, the road user will bear the costs in terms of fuel consumption, health and safety, and time. Additionally, surface cracks, rutting or reduced skid resistance are among the most common defects caused by an increase in the volume and intensity of traffic moving on highways [10]. Modified asphalt has played a vital role in reducing pavement-related issues by enhancing thermo–physical characteristics, which have an impact on the mixture’s final performance [11]. One of most popular ways to improve the performance of an asphalt mixture is to modify it with polymer [12,13]. In 1843, the first patent for incorporating polymers into asphalt was issued, and the first practical use of polymers in bitumen mix incorporation was found in the early 1990s [14].

**Figure 2:**
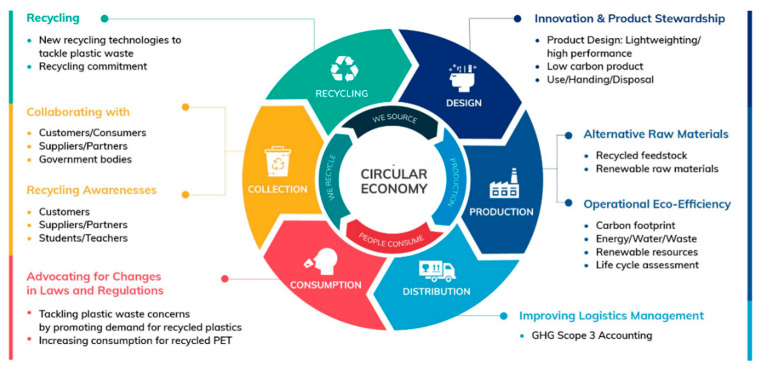
Circular Economy Cycle [19].

The circular economy concept is based on a policy that seeks (i) to overcome waste disposal problems by preserving and restoring existing products and (ii) utilize secondary raw global attention as a result of a basic restructuring of resource provision, production, and products while saving the cost of natural binder [15,16]. There are numerous definitions of CE both in the academic and gray literature [17,18]. We describe CE as “a model that has the potential to transform preserve, and reallocate materials, items, and goods back into the economy in an optimal way using as much as it is ecologically, functionally, socially, and cost effective”. Circular economy cycle has been shown in Figure 2 to understand the formation of steps of overall formation.

Similarly, it is necessary to keep in mind that not all types of waste are desirable for asphalt development [20]. The bulk of plastic bottles are composed of polyethylene terephthalate (PET). It degrades after a few uses and is therefore known as a serious issue due to the high cost of chemical treatment of PET [21]. As a result, chemical recycling is unlikely to be the ideal method for mitigating the crisis caused by massive amounts of PET disposal [10]. As a result, using PET as a mixed modifier in bituminous mix is a rational and cost-effective solution to this issue. A number of researchers found that PET can be used to increase rutting resistance in asphalt mix design [14]. According to another report, technology advances have made use of recycled materials to improve stiffness, strength, and surface appearance [22]. On the contrary, PET size of 1.18 mm showed a negative impact on rutting resistance and durability of the asphalt mixes when used as a modifier, as well as lower binder properties [23]. The softening point and viscosity of recycled PET bituminous mixes also increased significantly, according to the researchers [24]. A previous study found that adding 0.2–0.8% of PET content into the asphalt mix improved stability [25]. In terms of storage stability, polymers or plastics in asphalt mixture have problems maintaining their homogeneity. According to several studies, phase separation is a common issue [26,27,28]. Likewise, the use of different additives to PE with bituminous mixture has been shown to enhance homogeneity and also mixability, but with consequences on the binder characteristics and total price [29,30]. However, there is a still gap which exists in the literature on comprehensive lab testing to evaluate the performance of waste plastic at different percentage replacement and forms of plastics (PET and PE) used under different climatic conditions. Therefore, it is essential to explore the interactions of waste plastic in asphalt binder in terms of both physical and mechanical characteristics.

The primary objective of this work is to evaluate the potential reuse of secondary by-products as an additive. The physical and mechanical properties of polymer-based asphalt concrete with addition of two types of polymers (PET and PE) were tested and compared with control samples. Additionally, the effect of temperature for both plastic-modified asphalt mixtures were assessed in terms of Marshall stability and flow after 1 day and 30 days. This research provides a thorough analysis of the effects of both plastic wastes on asphalt mixtures, which can help in determining the potential for such products to be incorporated into the pavement sector.

## 2. Materials and Methods

**Figure 3:**
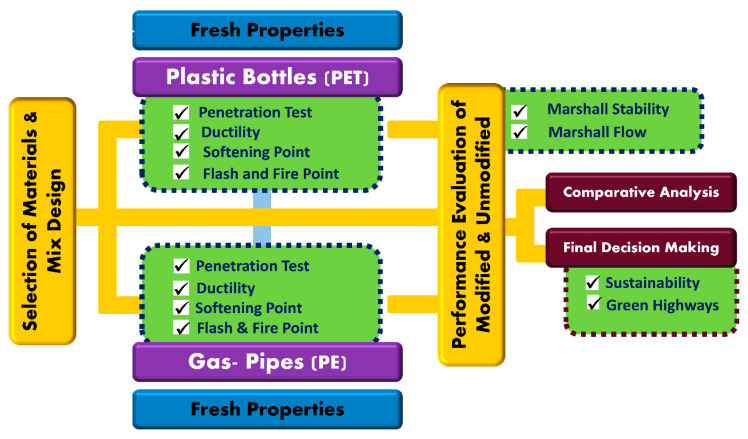
Flow Chart of Research Methodology.

In preparing the modified asphalt samples, standard procedure was performed. To determine the physical characteristics of the pavement constituent materials, multiple tests were carried out and compared with control specimens. Moreover, waste plastic-based modified asphalt concrete were tested and compared with unmodified asphalt concrete samples as per recommended specifications. The complete research methodology has been presented in Figure 3.

### 2.1. Materials

Asphalt with a penetration grade of 60–70 was used in this study. Different sizes of aggregate are used as a coarse and fine aggregate. Coarse aggregate of various sizes (19 mm, 12.5 mm, 9.5 mm and 4.75 mm) was used while the fine aggregate of <4.75 mm was utilized. The waste plastics such as crushed plastic bottles (PET) and gas pipe (PE) were collected from post-consumer and then passed through a washing process. These materials are further processed into small pieces by using crusher machines that are locally available in Pakistan. A portable gas burner was used for homogenous mixing of plastic waste with neat bitumen and to prepare the modified asphalt samples at standard temperature for mixing and testing.

### 2.2. Tests and Specifications for Asphalt Concrete Constituents

#### 2.2.1. Properties of Conventional binder

The properties of conventional binder comprising penetration, ductility, softening point and flash point test are analyzed as per ASTM specifications. In this study, a 60–70 grade of bitumen was used. The results of modified and pure binder are shown in Table 1.

#### 2.2.2. Properties of Aggregates

**Figure 4:**
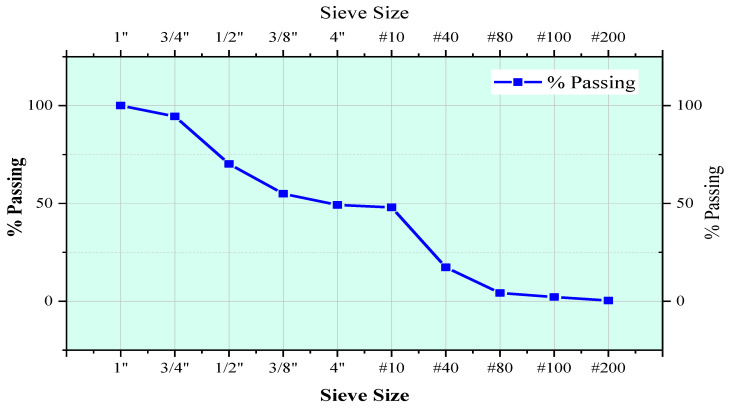
Aggregate Gradation Curve.

A sieve analysis test was performed to study and draw a particle size distribution curve for both aggregates, i.e., fine, and coarse aggregates as shown in Figure 4. This test is performed as per ASTM specification. The percentage passing of aggregate is then obtained to find out grain size distribution of aggregate used in this study. The properties of aggregate were analyzed and compressively tested in the laboratory. Results are presented along with their compliance standard and requirement in Table 2.

### 2.3. Mix Design and Preparation of Cake Samples

Marshall samples were cast with both modified and unmodified materials for better comparative analysis of the results. For this purpose, a total of 1200 gm of coarse aggregate and filler was collected carefully from the standard prepared mixture and then heated at a temperature ranging between 150 and 170 °C. Bitumen of penetration the grade of 60–70 was used and heated between temperatures of 160 and 170 °C. After that, hot aggregates and bitumen was mixed properly at a temperature of 165 °C which is designed for the 60–70 grade of bitumen. To achieve a homogenous mixing of both materials, asphalt mixer was used which is available in the Transportation Laboratory in PIET. At a temperature of 100 °C to 140 °C, the mixture is poured into the mold then tamped with 75 blows from both faces of the specimen using a standard hammer. After de-molding the samples with a hydraulic sample extractor, the sample was allowed to cool at room temp on a smooth, flat surface. The samples were put in a water bath which was thermostatically controlled for 30 to 40 min at a constant temperature of 60 °C for Marshall testing. According to the weight of the aggregates, the procedure was repeated to produce samples of 4.5% to 7.0% bitumen for calculation of optimum binder content. For preparation of modified samples, the same procedure was followed, and optimum binder content was replaced with both waste plastic content (PET and PE) in dosages of 0%, 5%, 10%,15% and 20% by total weight of binder. Moreover, waste plastic such as plastic bottles and gas pipes were shredded first and heated with pure binder to obtain a homogenous mixture. In the mixing phase for asphalt concrete preparation, the dry method was selected and used because it assumed more waste plastic to be incorporated in asphalt mixture. This method was also employed in this research [39,40]. For both PET- and PE-based asphalt mixtures, three samples at each percentage were prepared and then an average value were taken and compared with control samples (0%) for better comparative analysis of results. All samples were tested using Marshall stability machine.

## 3. Results and Discussions

### 3.1. Physical Performance of Plastic-Modified Bitumen

**Figure 5:**
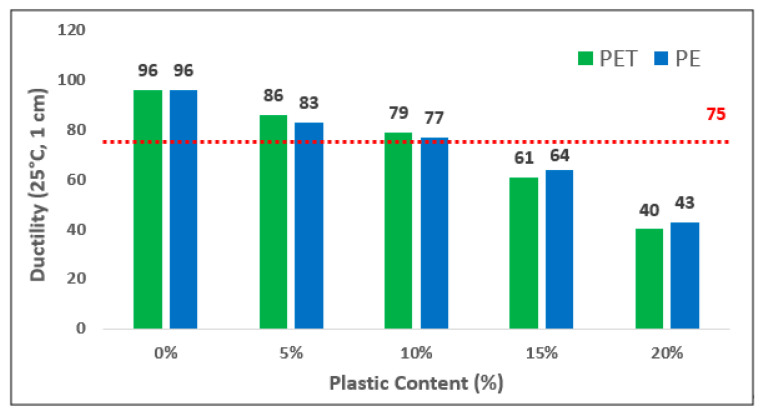
Ductility of Modified Bitumen versus different Plastic contents.

The physical performance of plastic-modified bitumen was analyzed by a penetration test, softening point, fire point and ductility tests as per standard specification. The penetration test was carried out using standard penetrometer apparatus. The standard Ring and Ball apparatus was used for determination of softening point while for flash point readings standard cleave land flash apparatus was used. Likewise, for ductility testing of both modified and unmodified binder standard ductility testing machine is used. The graph of ductility versus the plastic content, i.e., PET and PE utilized for the production of plastic-based asphalt mixture as shown in Figure 5. Overall, it can be seen that ductility value of plastic-modified bitumen was reduced with the addition of plastic contents. However, the ductility value of PET- and PE-modified bitumen up to 10% were >75 which is the minimum value as per the standard. After that, this value is decreased to reach to a peak of 40 and 43 cm by addition of 20% PET and PE in pure binder, respectively. With increased contents of both plastics, the ductility value of PET- and PE-based modified bitumen reduce because plastic addition makes it tough and difficult to flow. Hence, resistance to deformation under high temperature is improved by addition of plastic materials in modified asphalt mixtures.

**Figure 6:**
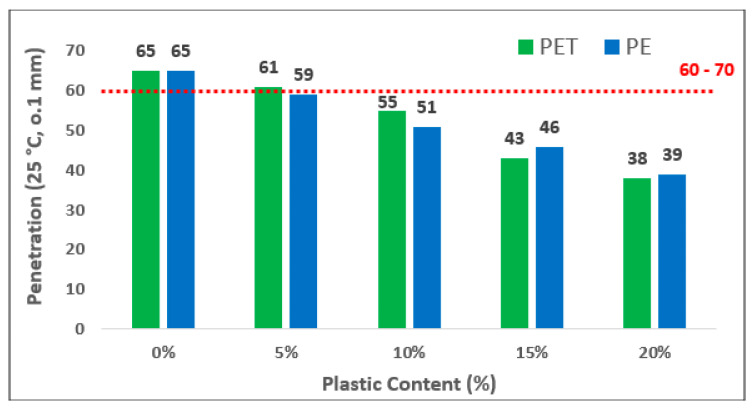
Penetration of Modified Bitumen versus different Plastic contents.

The bar chart illustrates the penetration value of both plastic contents in polymer-based modified bitumen in Figure 6. Overall, it can be seen that the maximum value of penetration stared at just over 5% and then falls dramatically to a value of 16% by 20% addition of plastic contents. A significant decrease in the value of penetration was due to addition of plastic contents which makes it more stiff and restricts its flow in varying climatic conditions which was also observed in these reports [41,42]. The reduction in penetration reading at 5%, 10%, 15% and 20% of PET content as compared to control sample was 6.2%, 11.8%,15.1% and 17%, respectively. Similarly, in the case of PPE contents, it was 11.0%, 12.7%,14.1% and 16% less as compared to neat binder.

**Figure 7:**
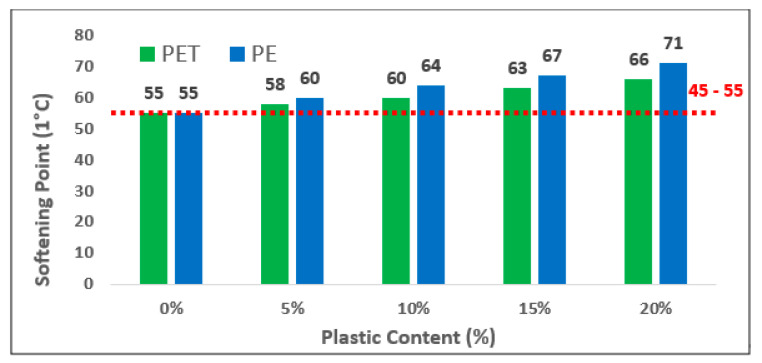
Softening Point of Modified Bitumen versus different Plastic contents.

The bar graph shows the softening point of plastic contents (PET and PE) in modified bitumen in Figure 7. The softening point value is measured in Centigrade (°C). It ranges between 45 and 55 °C for the penetration grade of 60–70 bitumen. Overall, it can be seen that the highest penetration value was recorded at 20% replacement of PE in pure bitumen. Moreover, a significant rise in penetration value was observed with the increase in plastic content in bitumen which was also observed in this report [41]. In case of PET addition, a similar trend was observed with varying percentages of plastic content in neat bitumen. The increase in softening point values was 5.2%, 8.1%, 13.7% and 17.7% at 5%, 10%,15% and 20% of PET replacement as compared to pure asphalt binder. Similarly, an increase of 9.4%, 14.1%, 18% and 23% was observed in case of PE contents as compared to the control binder. The higher value of softening point indicated that, at high temperature, asphalt binder is more stable, which is also mentioned in this report [42].

**Figure 8:**
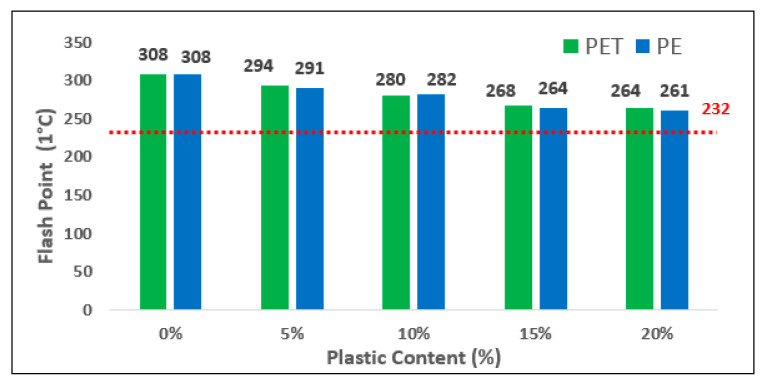
Flash Point of Modified Bitumen versus different Plastic contents.

The flash point value of both used plastic contents were measured as shown in Figure 8. From the bar chart, it can be seen that the flash point value is decreased with addition of plastic content in bitumen. However, the highest flash point reading is recorded at 0% which is a control sample. This value then falls steadily to reach a value of 264 and 261 in the case of both plastic materials. A similar trend of falling values in the graph was observed to greater than 232, which is the minimum required value as per specifications.

In conclusion, ductility and penetration of modified asphalt bitumen are improved with rise of waste plastic contents for both PE and PET. On the other hand, flash point and softening point are heightened with change in plastic contents of both PE and PET materials.

### 3.2. Mechanical Performance of Plastic-Modified Bitumen

**Figure 9:**
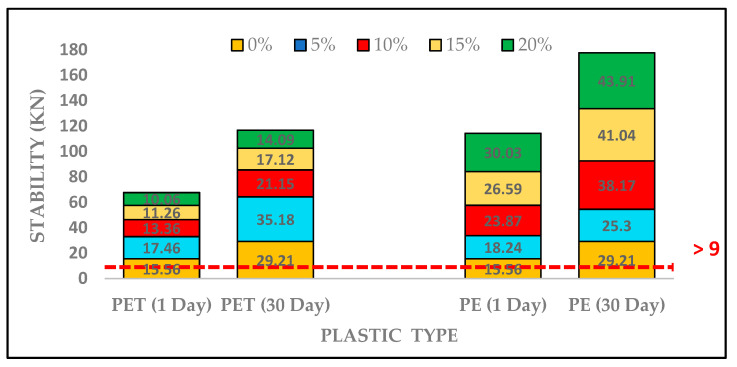
Stability of Plastic-Modified asphalt concrete under different testing days.

The Marshall stability values for PET- and PE-based asphalt mixture at 1 day and 30 day are presented in Figure 9. The stability value for both plastic contents at varying percentages were found to increase. The stability value improved with increasing in waste plastic contents for PET content and found 17.46, 13.36, 11.26 and 10.06 KN at 5%, 10%, 15%, and 20% replacement of PET contents, respectively. Additionally, the stability values of PE-based asphalt mixtures enhanced consistently from 18.24, 23.87, 26.59 and 30.03, respectively, after 1 day testing. The maximum stability value for PET content was observed at 5%. However, stability values obtained from PE-based asphalt mixture were greater as compared to PET-based asphalt mixture due to wider dispersion of gas pipes (PE) in PMB, which indicates more stiffness as well as increased stability.

Similarly, the Marshall stability values of PMB for PET and PE after 30 days of testing were found to increase. It was observed that addition of PET and PE in asphalt mixture makes it more stable under cyclic loading conditions. However, stability values after 30 days of testing were superior to those of PET content at similar percentage dosages.

**Figure 10:**
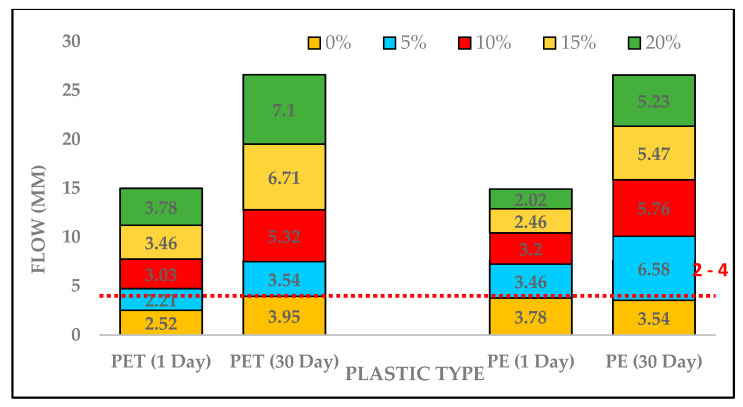
Flow of Plastic-Modified asphalt concrete on different testing days.

Flow values for PET-based asphalt concrete were elevated with increasing plastic contents as shown in Figure 10. Flow of 3.78 was observed at 20% replacement of PET which is higher than as compared to the control sample, but it was within the standard range. Similarly, a considerable increase in flow values of PMB for PET at 30 days were recorded. Additionally, flow values of PE-based asphalt mixture was within standard range after 1 day testing. On the other hand, flow values for PE content after 30 days testing was higher as compared to the control sample.

In summary, with a ramp up in plastic content, PET- and PE-modified asphalt concrete showed some similarities, and also some variations in their characteristics. We also found that PET and PE asphalt mixtures must be carefully prepared in order to achieve the desired physical and mechanical properties of PMB.

## 4. Conclusions

This study highlighted the significance of eco-friendly infrastructure projects in our pursuit to achieve sustainability in 21st century. Our work has suggested different waste materials, including plastic waste, which are suitable for various perspectives of road development. There is a need for cooperation among all investors and policymakers at all levels of government in order to allocate resources, as well as legislation, supervision, and compliance, for research and innovation to promote sustainable road infrastructure in developing nations. The findings of this research can be useful for policy decisions necessary to achieve a circular economy. Plastic waste could be referred to as the most appropriate waste potential treatment based on performance to achieve better environmental advantages in terms of resource usage.

- Additionally, this research investigated the effects of waste plastic materials, i.e., plastic bottles (PET) and gas pipes (PE) in asphalt mixtures used for road construction. The following conclusions were found from experimental works:
- Higher stability values were observed with increasing plastic content for both PET and PE waste materials. PE-based modified asphalt concrete showed superior strength properties as compared to PET-based modified asphalt mixtures. It showed that plastic type can be used for making new roads.
- The use of PET and PE waste plastics seems to cause reduction in readings of penetration and ductility values but causes increase in flash point and softening point values.
- In order to strengthen the reuse of plastic waste (PET and PE) by road engineering firms in building and rehabilitation of road pavements, aggressive guidelines, supervision, and support to encourage its adoption are desired, especially in the developing states.
- A successful collection of waste systems ought to be set in place by waste management agencies in order to facilitate the separate collection of waste plastic as well, to resolve this issue in an effective way.
- The use of waste plastic-modified asphalt mixtures in road construction will greatly boost the service life of our highways with positive benefits as well as help to mop up many million metric tons of waste plastic from landfill.

## Figures and Tables

**Table 1:** Physical properties of asphalt binder.

Parameter	Test Method	Avg. Values	Standard Range
Penetration @25 °C (0.1 mm)	ASTM D5-97 [31]	65	60–70
Softening point (°C)	ASTM D36	55	40–55
Flash Point (°C)	ASTM D92-16b [32]	308	232 min
Ductility 25 °C (cm)	ASTM D113-17 [33]	96	>75

**Table 2:** Properties of aggregates used.

Properties	Standard	Result	Requirement
**Coarse Aggregate**			
Aggregate Impact Test	BS 812-12:1990 [34]	21.33%	<27%
Los Angeles Abrasion Test	ASTM C131	27%	<35%
Aggregate Crushing Test	BS 812-110:1990 [35]	23.21%	<30%
Water Absorption Test	ASTM C127	1.90%	<2%
Specific Gravity	ASTM C127-15 [36]	2.33	2–3
**Fine Aggregate**			
Water Absorption test	AASHTO T84 [37]	0.52%	<2
Soundness test	ASTM C88 [38]	8	<15

## Data Availability

Data will be available on suitable demand.

## References

[1] Kaur G., Uisan K., Ong K.L., Lin C.S. (2018). Recent trends in green and sustainable chemistry & waste valorisation: Rethinking plastics in a circular economy. Curr. Opin. Green Sustain. Chem..

[2] Geyer R., Jambeck J.R., Law K.L. (2017). Production, use, and fate of all plastics ever made. Sci. Adv..

[3] Wagner S., Schlummer M. (2020). Legacy additives in a circular economy of plastics: Current dilemma, policy analysis, and emerging countermeasures. Resour. Conserv. Recycl..

[4] Bond V. (2019). Public Procurement of Road Building Materials: Research into Recycled Content.

[5] Chen Y., Cui Z., Cui X., Liu W., Wang X., Li X., Li S. (2019). Life cycle assessment of end-of-life treatments of waste plastics in China. Resour. Conserv. Recycl..

[6] Jambeck J.R., Geyer R., Wilcox C., Siegler T.R., Perryman M., Andrady A., Narayan R., Law K.L. (2015). Plastic waste inputs from land into the ocean. Science.

[7] Sun L., Xin X., Ren J. (2017). Asphalt modification using nano-materials and polymers composite considering high and low temperature performance. Constr. Build. Mater..

[8] Moghaddam T.B., Karim M.R., Syammaun T. (2012). Dynamic properties of stone mastic asphalt mixtures containing waste plastic bottles. Constr. Build. Mater..

[9] El-Shafie M., Ibrahim I., El Rahman A.A. (2012). The addition effects of macro and nano clay on the performance of asphalt binder. Egypt. J. Pet..

[10] Moghaddam T.B., Soltani M., Karim M.R. (2014). Experimental characterization of rutting performance of polyethylene terephthalate modified asphalt mixtures under static and dynamic loads. Constr. Build. Mater..

[11] Malarvizhi G., Sabermathi R., Kamaraj C. (2015). Laboratory study on nano clay modified asphalt pavement. Int. J. Appl. Eng. Res..

[12] Airey G. (2004). Styrene butadiene styrene polymer modification of road bitumens. J. Mater. Sci..

[13] Lin P., Yan C., Huang W., Li Y., Zhou L., Tang N., Xiao F., Zhang Y., Lv Q. (2019). Rheological, chemical and aging characteristics of high content polymer modified asphalt. Constr. Build. Mater..

[14] Ahmadinia E., Zargar M., Karim M.R., Abdelaziz M., Ahmadinia E. (2012). Performance evaluation of utilization of waste Polyethylene Terephthalate (PET) in stone mastic asphalt. Constr. Build. Mater..

[15] Kakar M.R., Mikhailenko P., Piao Z., Bueno M., Poulikakos L. (2021). Analysis of waste polyethylene (PE) and its by-products in asphalt binder. Constr. Build. Mater..

[16] Shanmugam V., Das O., Neisiany R.E., Babu K., Singh S., Hedenqvist M.S., Berto F., Ramakrishna S. (2020). Polymer recycling in additive manufacturing: An opportunity for the circular economy. Mater. Circ. Econ..

[17] Kirchherr J., Reike D., Hekkert M. (2017). Conceptualizing the circular economy: An analysis of 114 definitions. Resour. Conserv. Recycl..

[18] MacArthur E. (2013). Towards the circular economy. J. Ind. Ecol..

[19] INDORAMA (2020). Circular Economy. https://sustainability.indoramaventures.com/en/sustainability-knowledge-sharing/circular-economy/landing-page.

[20] Ismail Z.Z., Al-Hashmi E.A. (2008). Use of waste plastic in concrete mixture as aggregate replacement. Waste Manag..

[21] Li Y., White D.J., Peyton R.L. (1998). Composite material from fly ash and post-consumer PET. Resour. Conserv. Recycl..

[22] Tam V.W., Tam C.M. (2006). A review on the viable technology for construction waste recycling. Resour. Conserv. Recycl..

[23] Sojobi A.O., Nwobodo S.E., Aladegboye O.J. (2016). Recycling of polyethylene terephthalate (PET) plastic bottle wastes in bituminous asphaltic concrete. Cogent Eng..

[24] Abdelaziz M., Mohamed Rehan K. Rheological evaluation of bituminous binder modified with waste plastic material. Proceedings of the 5th International Symposium on Hydrocarbons & Chemistry (ISHC5).

[25] Soltani M., Moghaddam T.B., Karim M.R., Baaj H. (2015). Analysis of fatigue properties of unmodified and polyethylene terephthalate modified asphalt mixtures using response surface methodology. Eng. Fail. Anal..

[26] Zani L., Giustozzi F., Harvey J. (2017). Effect of storage stability on chemical and rheological properties of polymer-modified asphalt binders for road pavement construction. Constr. Build. Mater..

[27] Liang M., Xin X., Fan W., Wang H., Jiang H., Zhang J., Yao Z. (2019). Phase behavior and hot storage characteristics of asphalt modified with various polyethylene: Experimental and numerical characterizations. Constr. Build. Mater..

[28] Liang M., Sun C., Yao Z., Jiang H., Zhang J., Ren S. (2020). Utilization of wax residue as compatibilizer for asphalt with ground tire rubber/recycled polyethylene blends. Constr. Build. Mater..

[29] Fang C., Yu R., Zhang Y., Hu J., Zhang M., Mi X. (2012). Combined modification of asphalt with polyethylene packaging waste and organophilic montmorillonite. Polym. Test..

[30] Padhan R.K., Sreeram A. (2018). Enhancement of storage stability and rheological properties of polyethylene (PE) modified asphalt using cross linking and reactive polymer based additives. Constr. Build. Mater..

[31] (1997). ASTM D5-97, Standard Test Method for Penetration of Bituminous Materials.

[32] (2016). ASTM D92-16b, Standard Test Method for Flash and Fire Points by Cleveland Open Cup Tester.

[33] (2017). ASTM D113-17, Standard Test Method for Ductility of Asphalt Materials.

[34] BS 812-12: 1990 (1990). Testing Aggregates in Method For Determination of Aggregate Impact Value (AIV).

[35] BS 812-110: 1990 (1990). Methods for Determination of Aggregate Crushing Value (ACV).

[36] (2015). ASTM C127-15, Standard Test Method for Relative Density (Specific Gravity) and Absorption of Coarse Aggregate.

[37] AASHTO-T84 (2013). Specific Gravity and Absorption of Fine Aggregate.

[38] (2018). ASTM C88/C88M-18, Standard Test Method for Soundness of Aggregates by Use of Sodium Sulfate or Magnesium Sulfate.

[39] Ahmadinia E., Zargar M., Karim M.R., Abdelaziz M., Shafigh P. (2011). Using waste plastic bottles as additive for stone mastic asphalt. Mater. Des..

[40] Moghaddam T.B., Soltani M., Karim M.R. (2014). Evaluation of permanent deformation characteristics of unmodified and Polyethylene Terephthalate modified asphalt mixtures using dynamic creep test. Mater. Des..

[41] Essawy A., Saleh A.M., Zaky M.T., Farag R.K., Ragab A.A. (2013). Environmentally friendly road construction. Egypt. J. Pet..

[42] Fang C., Liu X., Yu R., Liu P., Lei W. (2014). Preparation and properties of asphalt modified with a composite composed of waste package poly (vinyl chloride) and organic montmorillonite. J. Mater. Sci. Technol..

